# Diagnostic accuracy assessment of molecular prediction model for the risk of NAFLD based on MRI-PDFF diagnosed Chinese Han population

**DOI:** 10.1186/s12876-021-01675-y

**Published:** 2021-02-25

**Authors:** Qing Zhang, Yueli Zhu, Wanjiang Yu, Zhipeng Xu, Zhenzhen Zhao, Shousheng Liu, Yongning Xin, Kuirong Lv

**Affiliations:** 1 Department of Radiology, Qingdao Municipal Hospital, 5 Donghaizhong Road, Qingdao, 266071 Shandong Province China; 2 Department of Infectious Disease, Qingdao Municipal Hospital, 1 Jiaozhou Road, Qingdao, 266011 Shandong Province China; 3 Clinical Research Center, Qingdao Municipal Hospital, 5 Donghaizhong Road, Qingdao, 266071 Shandong Province China

**Keywords:** Non-alcoholic fatty liver disease, MRI-PDFF, Molecular prediction model

## Abstract

**Background:**

Several molecular prediction models based on the clinical parameters had been constructed to predict and diagnosis the risk of NAFLD, but the accuracy of these molecular prediction models remains need to be verified based on the most accurate NAFLD diagnostic method. The aim of this study was to verify the accuracy of three molecular prediction models Fatty liver index (FLI), NAFLD liver fat score system (NAFLD LFS), and Liver fat (%) in the prediction and diagnosis of NAFLD in MRI-PDFF diagnosed Chinese Han population.

**Patients and methods:**

MRI-PDFF was used to diagnose the hepatic steatosis of all the subjects. Information such as name, age, lifestyle, and major medical histories were collected and the clinical parameters were measured by the standard clinical laboratory techniques. The cut-off values of each model for the risk of NAFLD were calculated based on the MRI-PDFF results. All data were analyzed using the statistical analysis software SPSS 23.0.

**Results:**

A total of 169 subjects were recruited with the matched sex and age. The ROC curves of FLI, NAFLD LFS, and Liver fat (%) models were plotted based on the results of MRI-PDFF. We founded that the accuracy of FLI, NAFLD LFS, and Liver fat (%) models for the prediction and diagnosis of NAFLD were comparative available in Chinese Han population as well as the validity of them in other ethnics and regions.

**Conclusions:**

The molecular prediction models FLI, NAFLD LFS, and Liver fat (%) were comparative available for the prediction and diagnosis of NAFLD in Chinese Han population. MRI-PDFF could be used as the golden standard to develop the new molecular prediction models for the prediction and diagnosis of NAFLD.

## Introduction

Non-alcoholic fatty liver disease (NAFLD) has become the most prevalent chronic liver disease over the past several years, and the average prevalence of NAFLD approximately 25% in the world, and even over the 30% in some districts [1–3]. The spectrum of NAFLD ranges from non-alcoholic fatty liver (NAFL), non-alcoholic steatohepatitis (NASH), fibrosis, cirrhosis, and even the hepatocellular carcinoma [4, 5]. Accurate predicating and diagnosing the risk of NAFLD and taking actions to prevent the progression of NAFLD shows great significance. Liver biopsy remains is the diagnostic gold standard of NAFLD, but the clinical application of liver biopsy is not extensive acceptable due to some defects such as invasiveness, sample error, and operator-dependent [6, 7]. Some noninvasive imaging diagnosis methods such as ultrasonography, computed tomography and controlled attenuation parameter had been conventional used in clinical diagnosis of NAFLD, but the accuracy could match to biopsy and need to be improved [8–10].

Magnetic resonance imaging derived proton density fat fraction (MRI-PDFF) is a newly developed and abundant applied NAFLD diagnose method recently years which can assess the liver fat over the entire liver accurately and MRI-PDFF possesses the higher repeatability and reproducibility [8, 11–13]. Imajo et al. reported that MRI-PDFF could accurate classify the grades of steatosis in biopsy-proven NAFLD patients [14]. Subsequently, Middleton et al. reported that MRI-PDFF has the high accuracy to both classify and predict histological steaotsis grade and change in histological steatosis grade in children with NAFLD [15]. A meta-analysis conducted by Gu et al. suggested that MRI-PDFF has excellent diagnostic value for assessment of hepatic fat content and classification of histologic steatosis in NAFLD patients [16].

In addition to the biopsy and imaging diagnostic methods, some molecular prediction models had been constructed and used for the prediction of development risk of NAFLD and the hepatic fat content based on the clinical parameters. As early as 2006, Bedogni et al. built a hepatic steatosis predictor which named “Fatty liver index (FLI)” using the clinical parameters such as gender, age, ethanol intake, gammaglutamyl-transferase (GGT), body mass index (BMI) and so on [17]. In 2009, Kotronen et al. reported a NAFLD liver fat score system (NAFLD LFS) which could predict the fat score in liver according to the metabolic syndrome (MS), type 2 diabetes (T2D), fasting insulin, AST and ALT. In addition, Kotronen et al. developed a model to predict the liver fat percent based on the above factors [18]. Subsequently, many studies had diagnostic the risk of NAFLD and the fat content in liver according to the molecular prediction model [19–22], but the specificity and sensibility were remains need improved.

In fact, PDFF testing has not yet been used in medical activities due to the lack of machines and relevant clinical data. For the NAFLD patients who can’t be diagnosed by MRI-PDFF, the molecular prediction models could be regarded as the preferred tools to predict the development risk of NALFD. In this study, all the NAFLD patients and health controls that we recruited were subjected to the MRI-PDFF, and the FLI, NAFLD LFS, and Liver fat (%) models were used to calculate the liver fat score and liver fat percent in all the subjects. The diagnostic accuracy of FLI, NAFLD LFS, and Liver fat (%) models were investigated according to the results of MRI-PDFF.

## Patients and methods

### Participants

From March 2019 to August 2019, a total of 169 participants were recruited randomly from the Department of Gastroenterology and healthy examination center of Qingdao Municipal Hospital that including NAFLD patients who diagnosed by ultrasonography and health peoples. This study was conducted in strict compliance with the Declaration of Helsinki [23]. This study was approved by the Ethics Committee of Qingdao municipal hospital and the written informed consents were obtained from all Participants. All the subjects were pre-diagnosis by B-type ultrasonography. All the subjects were Chinese Han population, and the age and sex were matched. Subjects with the following cases were excluded: (1) excess alcohol consumption (males > 210 g/w, females > 140 g/w) or cigarettes; (2) accompanied with viral hepatitis, drug-induced hepatitis, autoimmune hepatitis, or other factors induced chronic liver diseases; (3) suffering from tumors or surgery in nearly 2 years.

### Clinical parameters collection

The basic information of each participant such as name, age, lifestyle, and major medical histories were collected by a standard questionnaire. Subjects with excess alcohol intake and other liver-related diseases were excluded from this study. The height, weight, waist circumference (WC) and hip circumference (HC) of each participant were measured by two professional inspectors. The body mass index (BMI) was calculated based on the equation as weight(kg)/height^2^(m^2^). The diastolic blood pressure and systolic pressure of each participant were measured twice with a 5 min interval and the average values were recorded. Each participant was subjected to an overnight fasting and the venous blood was sampled. The serum levels of aspartate aminotransferase (AST), alanine aminotransferase (ALT), gamma-glutamyltranspeptidase (GGT), triglyceride (TG), total cholesterol (TC), low-density lipoprotein (LDL), high-density lipoprotein (HDL) and fasting serum insulin were measured by the standard clinical laboratory techniques, respectively. The values of FLI, NAFLD LFS, and Liver fat (%) were calculated according to the formula in the Table 1.

**Table 1:** The calculation formula for FLI, NAFLD LFS and liver fat (%)

Score	Algorithm	References
FLI	FLI = (e^0.953*log^_e_^(triglycerides) + 0.139*BMI + 0.718*log^_e_^(GGT) + 0.053*waist circumference−15.745^)/(1 + e^0.953*log^_e_^(triglycerides) + 0.139*BMI + 0.718*log^_e_^(GGT) + 0.053*waist circumference−15.745)^*100	[17]
NAFLD LFS	NAFLD LFS = − 2.89 + 1.18 * metabolic syndrome (yes = 1 / no = 0) + 0.45*type 2 diabetes (yes = 2 / no = 0) + 0.15 * fasting serum insulin (mU/L) + 0.04 * AST(U/L)—0.94 * AST/ALT	[18]
Liver fat (%)	10^(− 0.805+0.282 * metabolic syndrome (yes = 1 / no = 0) + 0.078*type 2 diabetes (yes = 2 / no = 0) + 0.525 * lg(fs − insulin[mU/L]) + 0.521 * lg(fs − AST[U/L]) − 0.454 * lg(AST/ALT))^	[18]

BMI, body mass index; FLI, fatty liver index; GGT, gamma-glutamyltransferase; NAFLD, non-alcoholic fatty liver disease; NAFLD LFS, non-alcoholic fatty liver disease liver fat score; AST, aspartate aminotransferase; ALT, alanine aminotransferase

### MRI-PDFF examination

Each participant was subjected to the MRI-PDFF examination. Participants were scanned using a 3.0 T MRI Ingenia system (Philips Healthcare, Best, the Netherlands) with a six-echo mDixon-quant gradient-echo sequence, the filp angle was 3° and the time of repetition (TR) was 5 ms [24]. MR imaging analysis was performed by two trained image analysts who were blinded to this project. MR images were reviewed by the Osirix software and manually placed circular regions of interest (ROIs) in each of the nine Couinaud liver segments of the MR imaging PDFF maps in each participant. PDFF in each of the nine ROIs was recorded and the entire liver PDFF values were calculated as the mean of the nine PDFF values. Participants with a MRI-PDFF value ≥ 6.4% was diagnosed as the NAFLD patients [25].

### Diagnostic criteria of MS and T2D

Metabolic syndrome was diagnosed according to the diagnostic criteria of Chinese Diabetes Society, and three or more of the following items are sufficient for diagnosis: (1) WC ≥ 90 cm (male) and 85 cm (female); (2) FPG ≥ 6.1 mmol/L or 2-h PG levels ≥ 7.8 mmol/L after a 75 g oral glucose-tolerance test or have been diagnosed with diabetes; (3) blood pressure ≥ 130/85 mmHg or had been diagnosed with hypertension; (4) TG ≥ 1.7 mmol/L; 5) HDL < 1.04 mmol/L [26]. T2D was defined as typical symptoms of diabetes (polydipsia, polyuria, polyphagia, and weight loss) plus random plasma glucose ≥ 11.1 mmol/L, or FPG ≥ 7.0 mmol/L, or OGTT 2 h PG ≥ 11.1 mmol/L [26].

### Statistical analysis

All data were analyzed using the statistical analysis software SPSS 23.0. All the volunteers were categorized into the NAFLD group and health group according to the clinical characteristics and MRI-PDFF test. Qualitative data composition ratios were compared using chi-square analysis, and ages between NAFLD group and health group were compared using student t-test. The paired-samples t-test was used to assess the difference in predicted liver fat (%) and actual liver fat (%) detected by MRI-PDFF, while Spearman’s rank correlation test was used to assess the correlations of them. We used the receiver operating characteristic (ROC) analysis to redetermine the cut-off values of FLI, LFS, and liver fat (%) in diagnosing NAFLD. The sensitivity and specificity of cutoff value were calculated. "Youden index" was used to determine the optimal cutoff, Sensitivity—(1-specificity), and the maximum value of the index value is the optimal cutoff value. *P* < 0.05 was considered statistically significant.

## Results

### Clinical characteristics of included participants

According to clinical characteristics and MRI-PDFF results, participants were divided into two groups, NAFLD group (n = 109) and health group (n = 60). Characteristics and disease composition of NAFLD group and health group were shown in Table 2. There was no significant difference in age between the two groups (*P* > 0.05). The proportion of male patients with NAFLD was higher than male of the control group (*P* = 0.009). BMI value was significant higher in the NAFLD group (28.3 ± 4.1) than in the health group (25.1 ± 4.0) (*P* < 0.001). The proportion of patients with metabolic syndrome in NAFLD group (42.20%) was significantly higher than that in health group (18.33%) (*P* = 0.002). There was no significant difference in the composition of diabetic patients between the two groups (*P* > 0.05).

**Table 2:** Characteristics and disease composition of NAFLD and health groups

	NAFLD (n = 109)	Health group (n = 60)	t/χ^2^	*P* value
Age (mean ± SD), years	42.2 ± 12.8	42.0 ± 12.8	0.119	0.906
Male, n (%)	70 (64.22)	26 (43.33)	6.880	0.009
BMI (mean ± SD), kg/m^2^	28.3 ± 4.1	25.1 ± 4.0	0.478	< 0.001
T2D, n (%)	12 (11.01)	2 (3.33)	–	0.142
MS, n (%)	46 (42.20)	11 (18.33)	9.863	0.002

*BMI* body mass index, *NAFLD* non-alcoholic fatty liver disease, *T2D* type 2 diabetes, *MS* metabolic syndrome

Data were compared by t-test, fisher-test or chi-square analysis when appropriate. *P* < 0.05 was considered statistically significant

### NAFLD and fatty liver index (FLI)

**Fig. 1:**
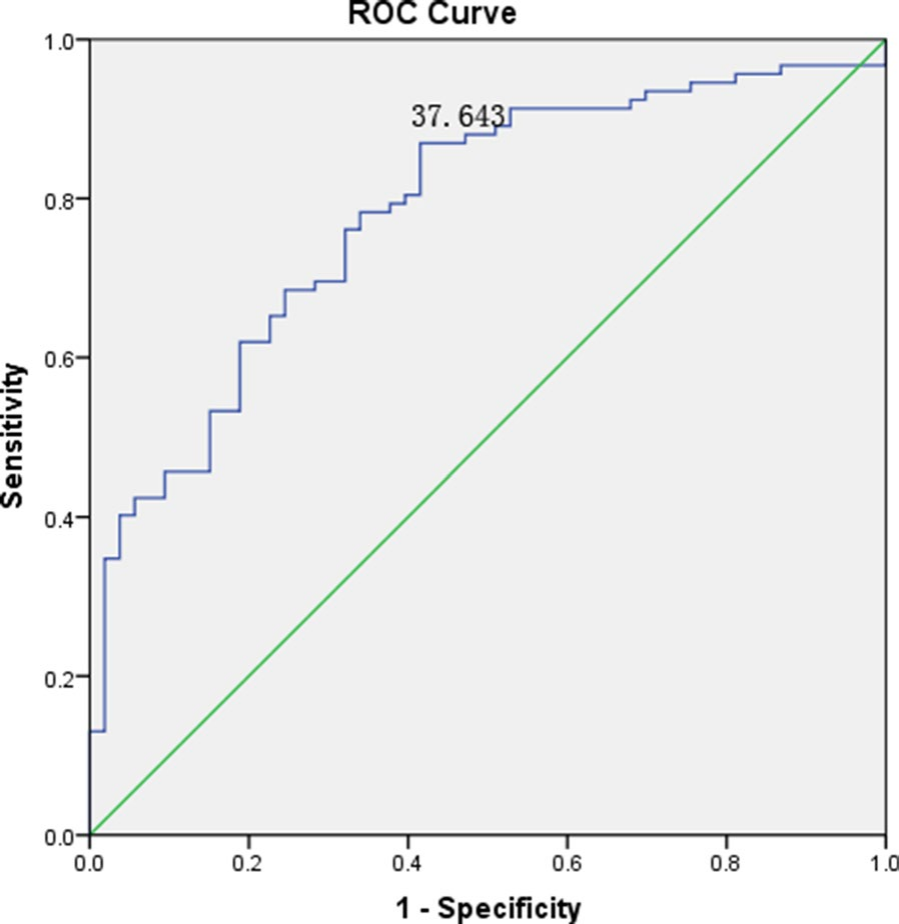
ROC curve of Fatty Liver Index model in MRI-PDFF diagnosed subjects. The AUROC is 0.78 (95% CI: 0.71–0.86). The optimal cut-off point (37.64) with sensitivity of 87.0% and specificity of 58.5% determined using the Youden index

In order to investigate the accuracy of FLI on the diagnosis of NAFLD, the ROC curve was plotted based on FLI score and MRI-PDFF diagnosis results (Fig. 1). Youden index was used to calculate the optimal cut-off value (cut-off ≥ 37.643), with a sensitivity of 87.0% and specificity of 58.5%. The area under the ROC curve (AUROC) was 0.78 (95% CI: 0.71–0.86). In the original literature [17], hepatic steatosis could be diagnosed when the FLI score ≥ 60. In addition, in our ROC curve, the FLI score 60 possesses a sensitivity of 59.8% and specificity of 81.1%. These results suggested that this molecular prediction model was comparative superior.

### NAFLD and NAFLD liver fat score (NAFLD LFS)

**Fig. 2:**
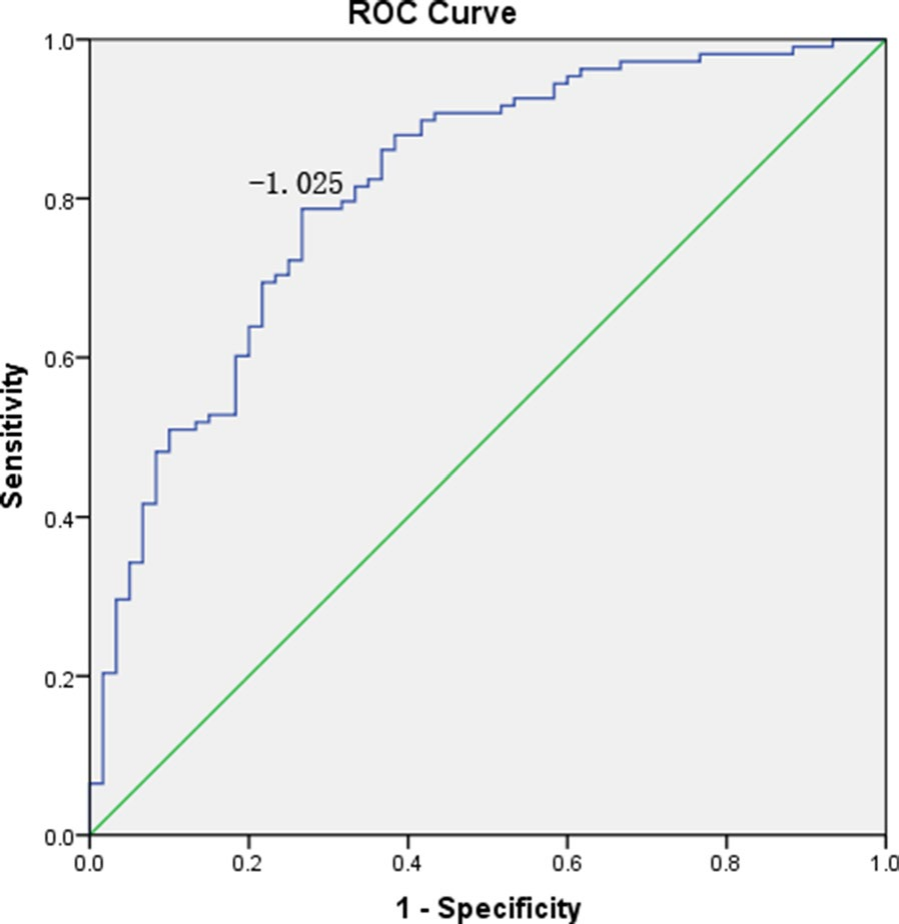
ROC curve of NAFLD liver fat score model in MRI-PDFF diagnosed subjects. The AUROC is 0.81 (95% CI: 0.74–0.88). The optimal cut-off point (−1.025) with a sensitivity of 78.7% and specificity of 73.3% determined using the Youden index

In order to investigate the accuracy of NAFLD LFS on the diagnosis of NAFLD, we plotted ROC curve based on NAFLD LFS and MRI-PDFF diagnosis results (Fig. 2). Youden index was applied to determine the optimal cut-off value for NAFLD, and we found that the NAFLD LFS values greater than -1.025 could predict the development of NAFLD (sensitivity = 78.7%, specificity = 73.3%). The AUROC was 0.81 (95% CI: 0.74–0.88). The optimal cut-off value in the original literature (LFS ≥ −0.640) [18] was substituted into this ROC curve and the corresponding sensitivity was 67.6% and specificity was 78.3%. When sensitivity (86%) was the same as that of the original text, the cutoff value was −1.4342, which was lower than the optimal cutoff value. These results suggested that this molecular prediction model was comparative superior.

### NAFLD and liver fat (%)

**Fig. 3:**
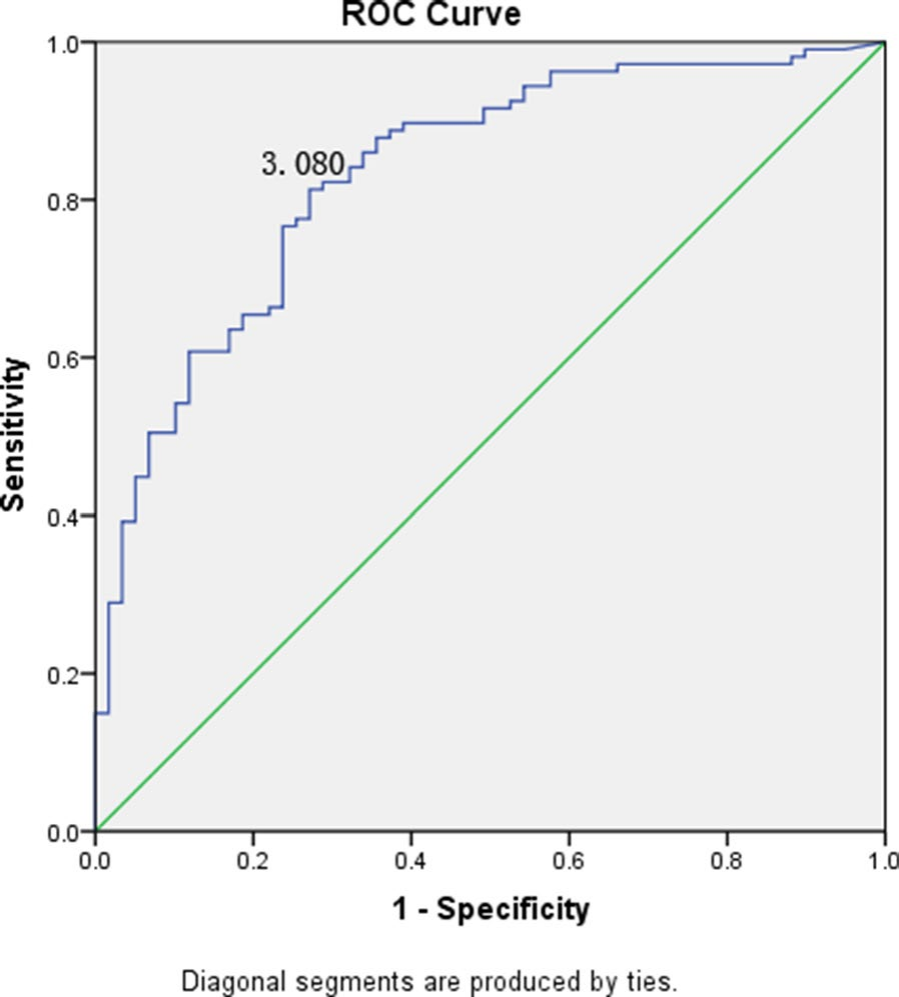
ROC curve of Liver Fat (%) model in MRI-PDFF diagnosed subjects. The AUROC was 0.83 (95% CI: 0.77–0.90). Youden index was used to calculate the optimal cut-off value (cut-off ≥ 3.080), with a sensitivity of 81.3% and specificity of 72.9%

Kotronen A et al. developed a formula to predict the liver fat percentage. In this study, the paired-samples t-test was used to assess the difference in predicted liver fat percentage and actual liver fat percentage which was detected by MRI-PDFF, and the significant difference of the liver fat between the two results was observed (*t* = 10.357, *P* < 0.001). Spearman’s rank correlation test was used to assess the correlation of them, as the results shown, the correlation coefficient was 0.649 (*P* < 0.001), which indicated that the predicted value of liver fat by the formula is moderately correlated with the actual value. We plotted ROC curve based on predicted liver fat percentage and NAFLD diagnosis results (Fig. 3), and the AUROC was 0.83 (95% CI: 0.77–0.90). Youden index was used to calculate the optimal cut-off value (cut-off ≥ 3.080), with a sensitivity of 81.3% and specificity of 72.9%.

## Discussion

NAFLD is defined as the excess accumulation of fat in the liver and tightly associated with the obesity, T2D, MS, insulin, and dyslipidemia [18, 27]. Hepatic steatosis is the early stage of NAFLD and could progress to the NASH [28]. Accurate prediction and diagnosis of NAFLD in the early stage will take good effects on the prevention of NAFLD progression and the development of various complications. In recent years, MRI-PDFF had been used to diagnose the hepatic steatosis in several countries and the reliable accuracy, sensitivity and specificity had been verified [14, 15, 25, 29]. Besides the imaging and pathological diagnosis, some molecular prediction models based on the clinical parameters, genetics information, and serum biochemical factors had been constructed for the prediction of the risk of NAFLD frequently [30–32]. Chinese people's diet structure, obesity type and other susceptible factors were not exactly the same as those of other regions, therefore the sensitivity and specificity of the models were remain worth discussed based on the most accurate NAFLD diagnostic method such as MRI-PDFF in different ethnics and regions. For suspected NAFLD, biopsy was recommended for confirmation. Since biopsies were invasive, we need a reliable, safe diagnostic alternative to biopsy. In this study, we performed the MRI-PDFF examination as the NAFLD diagnostic gold standard to verify the diagnostic value of NAFLD molecular prediction model FLI, NAFLD LFS and Liver fat (%) in Chinese Han population that had been reported previously [17, 18]. We found that these NAFLD prediction models possess the relatively good diagnostic sensitivity and specificity for the risk of NAFLD. Meanwhile, these molecular prediction models could be optimized for the optimal diagnostic accuracy.

BMI is a tightly correlative factor with the risk of NAFLD and could be regarded as the indicator for the development of NAFLD [33, 34]. In our study, we divided all the participants into NAFLD group and health group with according to the MRI-PDFF examination results. The average BMI value in NAFLD group was significantly higher than health group, which is accord with the classical conclusion. In addition, the prevalence of T2D and MS in NAFLD group was also higher than in health group, which suggested that NAFLD patients possess the high risk of T2D and MS.

Bedogni et al. reported the fatty liver index which was a predictor of hepatic steatosis in the general population. According to the model, a FLI < 30 could be rule out hepatic steatosis with the sensitivity was 87% and a FLI ≥ 60 could be rule in hepatic steatosis with the specificity 86% [17]. In our study, we used this model to predict the risk of NAFLD in the MRI-PDFF verified population, and we found that the optimal cut-off value was FLI ≥ 37.64 with a sensitivity of 87.0% and specificity of 58.5%. In addition, the FLI score 60 possesses a sensitivity of 59.8% and specificity of 81.1%. in our MRI-PDFF diagnosed population. The differences of the diagnostic sensitivity and specificity of FLI model verified by Bedogni et al. and us may contributed by the diagnostic criterion and methods, and the differences of ethnic and region, which may result in some biases in the NAFLD diagnosis. These results suggested that the accuracy of molecular prediction model could be improved when MRI-PDFF was selected as the NAFLD diagnostic golden standard. Kotronen et al. developed a NAFLD liver fat score (NAFLD LFS) model to screen NAFLD patients from general population with the available clinical and laboratory parameters. They found that the NAFLD could be confirmed with the sensitivity of 86% and specificity of 71% when the cutoff value was greater than −0.640 [18]. In this study, we predicted the risk of NAFLD using the NAFLD LFS model in the MRI-PDFF verified population, and the optimal cut-off value for NAFLD diagnosis was -1.025 with the sensitivity of 78.7% and specificity of 73.3%. When sensitivity was the same as that of the original text (86%), the cutoff value was -1.4342, which was lower than the optimal cutoff value. Although sensitivity was increased and cut-off value decreased, specificity was decreased. The diagnostic sensitivity and specificity was comparative, and the smaller differences may also result in by the diagnostic criteria and methods, and the differences of ethnic and region, which may lead to the misdiagnosis of NAFLD, and therefore the accuracy of the score was interfered. In addition to this, Hernando et al. found that although it could be corrected, the small PDFF biases observed across vendors [35].

In summary, we used MRI-PDFF as the NAFLD diagnostic gold standard to verify the diagnostic values of molecular prediction model in NAFLD patients. Our results suggested that the FLI, NAFLD LFS, and Liver fat (%) models were comparative accurate and could be regard as the useful tool to scan the NAFLD patients from general population in Chinese Han population. Especially, the accuracy of molecular prediction models could be improved when the MRI-PDFF was used as the diagnostic golden standard. This study reevaluated the three existing models and provided a more reliable truncation value for the Chinese population. This might help to assess the risk of NAFLD through routine physical examinations, saving medical resources and reducing the burden on patients. In future, more molecule prediction models which based on easily available clinical and laboratory data should be constructed according to accurate diagnosis of NAFLD by MRI-PDFF.

## Data Availability

The datasets used and/or analysed during the current study are available from the corresponding author on reasonable request.
